# The Impact of Ar or N_2_ Atmosphere on the Structure of Bi-Fe-Carbon Xerogel Based Composites as Electrode Material for Detection of Pb^2+^ and H_2_O_2_

**DOI:** 10.3390/gels10040230

**Published:** 2024-03-28

**Authors:** Carmen I. Fort, Mihai M. Rusu, Liviu C. Cotet, Adriana Vulpoi, Milica Todea, Monica Baia, Lucian Baia

**Affiliations:** 1 Faculty of Chemistry and Chemical Engineering, “Babes-Bolyai” University, Arany Janos 11, RO-400028 Cluj-Napoca, Romania; ioana.fort@ubbcluj.ro (C.I.F.); cosmin.cotet@ubbcluj.ro (L.C.C.); 2 Institute for Research-Development-Innovation in Applied Natural Sciences, “Babes-Bolyai” University, Fântânele 30, RO-400294 Cluj-Napoca, Romania; mihaimrusu@gmail.com; 3 Institute of Interdisciplinary Research in Bio-Nano-Sciences, “Babes-Bolyai” University, T. Laurean 42, RO-400271 Cluj-Napoca, Romania; adriana.lazar@ubbcluj.ro (A.V.); militodea@yahoo.com (M.T.); 4 Faculty of Physics, “Babes-Bolyai” University, M. Kogalniceanu 1, RO-400084 Cluj-Napoca, Romania; 5 Faculty of Medicine, “Iuliu Haţieganu” University of Medicine and Pharmacy, Victor Babeș 8, RO–400012 Cluj-Napoca, Romania

**Keywords:** carbon nanocomposites, bismuth, iron, lead detection, hydrogen peroxide detection

## Abstract

In this study, bismuth- and iron-embedded carbon xerogels (XG) were obtained using a modified resorcinol formaldehyde sol–gel synthesis method followed by additional enrichment with iron content. Pyrolysis treatment was performed at elevated temperatures under Ar or N_2_ atmosphere to obtain nanocomposites with different reduction yields (XGAr or XGN). The interest was focused on investigating the extent to which changes in the pyrolysis atmosphere of these nanocomposites impact the structure, morphology, and electrical properties of the material and consequently affect the electroanalytical performance. The structural and morphological particularities derived from X-ray diffraction (XRD), Raman spectroscopy, scanning electron microscopy (SEM), transmission electron microscopy (TEM), and X-ray photoelectron spectroscopy (XPS) measurements revealed the formation of the nanocomposite phases, mostly metal/oxide components. The achieved performances for the two modified electrodes based on XG treated under Ar or N_2_ atmosphere clearly differ, as evidenced by the electroanalytical parameters determined from the detection of heavy metal cations (Pb^2+^) or the use of the square wave voltammetry (SWV) technique, biomarkers (H_2_O_2_), or amperometry. By correlating the differences obtained from electroanalytical measurements with those derived from morphological, structural, and surface data, a few utmost important aspects were identified. Pyrolysis under Ar atmosphere favors a significant increase in the α-Fe_2_O_3_ amount and H_2_O_2_ detection performance (sensitivity of 0.9 A/M and limit of detection of 0.17 μM) in comparison with pyrolysis under N_2_ (sensitivity of 0.5 A/M and limit of detection of 0.36 μM), while pyrolysis under N_2_ atmosphere leads to an increase in the metallic Bi amount and Pb^2+^ detection performance (sensitivity of 8.44 × 10^3^ A/M and limit of detection of 33.05 pM) in comparison with pyrolysis under Ar (sensitivity of 6.47·10^3^ A/M and limit of detection of 46.37 pM).

## 1. Introduction

The detection of aqueous pollutants, such as heavy metal cations, is a global necessity due to their impact on human and animal health and the environment [1,2,3]. There is a continuous interest from the World Health Organization (WHO) [4] and the European Union [5] to evaluate heavy metal cation toxicity. Upgrades are sought for many techniques (atomic fluorescence spectrometry, atomic absorbance spectrometry, chemiluminescence, chemiresistive, electrochemical, and colorimetry detections) to improve not only the key parameters, such as sensitivity, selectivity, and detection limit, but also other requirements such as flexible usage, fast response, cost-effectiveness, and on-site measurements. Among these, the electrochemical technique is considered to be the most promising one in principle because it allows a strong adsorption and exhibits fast electron and mass-transfer kinetics and biocompatibility, as a result of the incorporation of nanomaterials into the electrode. To detect heavy metal cations, electrochemical sensors with interfaces such as nanoparticles (Au, Bi, etc.) [6,7], carbon based nanocomposite (CNTs [8], carbon aerogel (CA) [9,10], carbon xerogels [10,11,12], etc.), and metal oxides [6,13] have been developed. 

In this respect, it is worth emphasizing that our researches started years ago by intensively studying the electroanalytical performances of modified electrodes based on aerogel and xerogel carbon matrices, that were gradually modified with Bi (nanoparticles), Fe (oxide nanoparticles), or/and TiO_2_ (aerogel), by fixing of one parameter at a time (e.g., carbon matrix, metal type, concentration of metal doping, or pyrolysis temperature). Thus, Fe-based carbon aerogel was used for carbon paste-modified electrode preparation to detect H_2_O_2_ [9]. Later, the electrode matrix was changed from carbon paste electrode to glassy carbon electrode (GCE) [11], which correlated with a significant improvement in the material economy (from 10^−1^ g to 10^−5^ g) and the electrode sensitivity as well. As an electrode modifier, Bi-based CA matrix was used [11]. The important benefit of these electrode materials is that they do not imply the presence of difficult constituents, such as enzymes, other carbon-based nanomaterials (CNT, graphene, etc.), or noble metals, and involves a sol–gel preparation procedure, thus consuming very small amounts of electrode material and leading to competitive sensor performances [9,11]. Since one of the main goals was to eliminate an important step in the matrix synthesis (i.e., supercritical drying, which is a time- and cost-consuming process), our research focused on the comparison between Bi-modified carbon aerogel and Bi-modified carbon xerogel matrix as electrode material. Thus, the simultaneous detection of Cd^2+^ and Pb^2+^ cations using square wave anodic stripping voltammetry (SWASV) leads to important performance features [10]. Then, taking into consideration the results acquired in the nanocomposite application field, a combination of both Bi and Fe in the carbon xerogel matrix was studied [13,14,15]. Dopant metal concentration [13] and pyrolysis temperature [14,15] were carefully investigated. The next step was the addition of TiO_2_ to Bi-Fe-carbon xerogel based-composites (XG, given the potential of the newly added component to extend the material functionality (i.e., photocatalysis)). All these approaches were essential from the perspective of obtaining nanomaterials with proper characteristics that can be applied to prepare inks for screen printed electrodes or involved in patterning deposition procedures based on lithographic techniques. Moreover, the intensively studied methodology and adaptability of sol–gel chemistry open innovative directions for the cost-effective synthesis of electrode materials for the construction of miniaturized electrochemical sensors, allowing such devices to be used for simple and environmentally friendly sensing. The variation in Fe concentration was also investigated, and it was found that the increase in the Fe content led to significant changes at the nanoscale [13]. The achieved electroanalytical parameters proved that the increase in Fe content in the XG nanocomposite improves its performances for H_2_O_2_ detection and decreases the Pb^2+^ detection efficiency. However, it is important to note that the sensing performances for Pb^2+^ (1.24 pM) [13] remain well below the maximum allowed concentration in drinking water (48 nM). Once again, it is highlighted that this material is well-suited for these two different applications and may represent a starting point for solving challenging situations where heavy metal cations and biological environments coexist. A possible limitation of the prepared modified electrode is the separate determination of heavy metals and H_2_O_2_ due to the differences in the experimental conditions that are needed.

Continuing our research in the field of nanocomposites materials [9,10,11,12], we proposed this time to investigate the extent to which changes in the pyrolysis atmosphere of XG nanocomposite will impact the structure, morphology, and electrical properties of material and will consequently affect electroanalytical performances. The choice in treatment temperature, dwell time, and pyrolysis atmosphere has led to different electric properties of the carbon support [16] and embedded nanoparticles with oxide [12] and/or metallic nature [9,17]. Therefore, to obtain carbon-based nanocomposites with different metal oxide reduction yields, we propose to apply a pyrolysis step at 1050 °C under Ar or N_2_ flow. The reducing gas was selected by considering its frequent use in the pyrolysis of carbon composites and keeping in mind that Ar is a noble gas and N_2_ is inert until molecular dissociation takes place. Hence, reactions with oxygen, carbon, hydrogen, and metal surfaces can occur, leading to different reduction yields [18,19]. Higher thermal treatment leads to the generation of nanocomposites in an advanced reduction/incipient graphitization stage and improved electrochemical response [14,15]. The changes in the carbon support and the electroactive surface of both Bi- and Fe-based nanoparticles are characterized by X-ray diffraction (XRD), Raman spectroscopy, scanning and transmission electron microscopies (SEM and TEM), and X-ray photoelectron spectroscopy (XPS) measurements. The beneficial effect of both Fe and Bi addition and the pyrolysis atmosphere (Ar or N_2_) was explained based on morphological, structural, and electrical characterization. Additionally, correlations between the obtained results and those achieved from electroanalytical measurements for the detection of heavy metal cations (Pb^2+^, by square wave voltammetry (SWV) technique) or biomarkers (H_2_O_2_, by amperometry) were assessed. One should note that none of our previous studies reported the analysis of samples with such a high amount of Fe. Additionally, after applying a pyrolytic treatment under N_2_ and Ar atmosphere, the correlation between the morpho-structural findings and the electrochemical features was not assessed. Thus, by choosing the atmosphere in which the pyrolysis of the material is carried out (N_2_ or Ar), it becomes possible, depending on the intended application, to synthesize the proper material capable of developing the desired properties.

## 2. Results and Discussion

### 2.1. Morphological and Structural Analyses

Nanoporous carbon xerogels with embedded Bi and Fe and a conserved monolithic shape were obtained using an incipient sol–gel route through which the metal precursors salts were finely dispersed into the cross-linked polymer matrix as described elsewhere [12]. The presence of the precursor Bi salt (i.e., Bi(NO_3_)_3_·5H_2_O) dissolved in glycerol formal that is adjusted with ammoniac/acetic acid mixture could be considered as having a catalytic action for the polycondensation reaction of resorcinol (i.e., the main polymer precursor) with formaldehyde. Moreover, the presence of metals in the resorcinol–formaldehyde gel structure provides a lower probability for the occurrence of a morpho-structural collapse during ambient drying and pyrolytic steps of the ternary-component xerogel synthesis pathway. In our previous studies, we have observed an increased performance in H_2_O_2_ detection at higher concentrations of Fe precursor [12]. The addition of a supplementary Fe impregnation step is investigated here as a new strategy for Fe enrichment of the nanocomposite because a further increase in the Fe precursor concentration in the initial co-synthesis route may lead to a disturbance of the sol–gel polycondensation reaction (i.e., a higher amount of the involved solvent for the reaction of resorcinol and formaldehyde could be involved in iron salt dissolution). Also, by investigating binary C-Fe systems, we have observed the reduced iron states and also noted that Fe assisted the growth of graphitic carbon nanostructures after pyrolysis at elevated temperatures [9]. It is worth noting that after pyrolysis, the newly obtained nanocomposites continued to preserve their monolithic shape. Even though the samples exhibited a clear response to magnetic fields, their magnetic properties were not analyzed in this study. Bi-Fe-carbon xerogel based composites pyrolyzed separately under N_2_ and Ar atmosphere are further denoted as XGN and XGAr, respectively.

**Figure 1:**
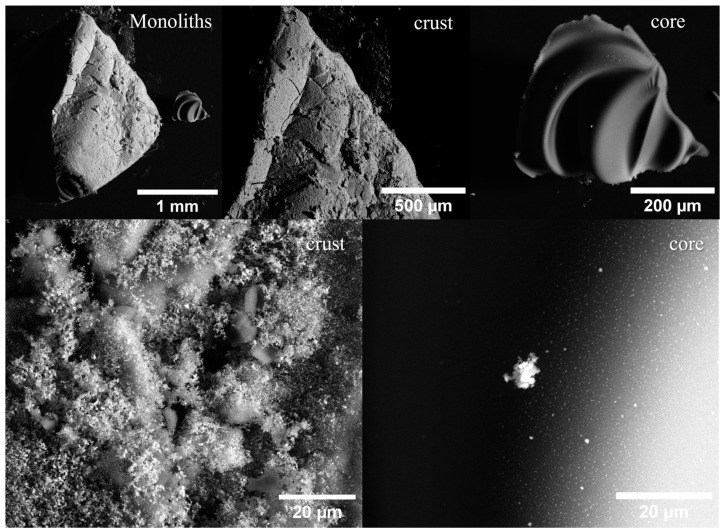
SEM micrographs obtained from XGN composite reveal both the surface of the obtained grains and their core exposed through grinding.

In a previous study, we investigated similar nanocomposites obtained without the additional impregnation, but followed by a pyrolysis step. It was found that nanoparticles based on Bi and Fe are formed mainly inside the carbon support. The quantification results of the EDX spectra acquired from the surface of the grains were expressed as a C:O:Bi:Fe ratio of 94.22:5.20:0.42:0.15 in at%. The consequences of the extra impregnation step followed by pyrolysis under inert gas conditions are reflected in the SEM micrographs presented in Figure 1.

Visual inspection shows that the impregnated monoliths are covered by a dark red/orange layer. Fractal topology is visible at microscales, revealing nano-sized features and an elemental composition (C:O:Bi:Fe at%) of 60.60/24.96/0.03/14.41 (observed for XGN sample). This mainly indicates that the iron oxide phases are abundantly grown at the interface between the material and the pyrolysis atmosphere. Upon fracturing, the core of the nanocomposite is exposed, and nanoparticles are revealed as bright centers grown inside the carbon ultrastructure. The EDX elemental composition obtained from core specific regions is 96.19/0.70/0.35/2.75. In comparison to the co-synthesized system, it can be observed that the core also exhibits higher Fe content. This result suggests that the impregnation route would greatly affect not only the surface of the pre-obtained nanocomposite, but also its volume mainly via bulk diffusion through the porous network. 

**Figure 2:**
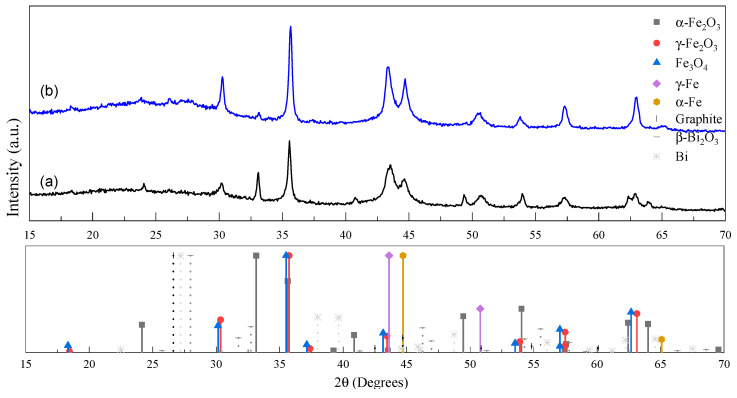
XRD patterns and reference signals for (a) XGAr and (b) XGN nanocomposites together with the reflections given by the crystalline structures, as these are indicated in the figure legend (according to the database).

The structural phase composition of the nanocomposite xerogel is investigated using XRD as presented in Figure 2 and Table 1. The broad signal between 2θ = (15, 40) is specific to vitreous carbon phases found in carbon xerogel composites.

Regarding non-ferrous components, turbostratic graphite (AMCSD 0000049) and Bi phases (both in metallic -AMCSD 0011254- and oxidized state -AMCSD 0010069-) are marked only as minute contributions at 2θ = 26.05° and 2θ = 27.05–27.90°, respectively. The main reflections indicate the coexistence of multiple Fe-based structures in both investigated samples: maghemite (γ-Fe_2_O_3_—AMCSD 0007898)/magnetite (Fe_3_O_4_—AMCSD 0000945), hematite (α-Fe_2_O_3_—AMCSD 0000143), and the metallic phases α-Fe (AMCSD 0000670) and γ-Fe (AMCSD 0019406). A significant decrease in the α-Fe_2_O_3_ contribution is observed that mostly differentiates the XGN sample from its counterpart. The results of the quantitative analysis of Fe-based phases (presented in Table 1) indicate that the treatment under Ar atmosphere leads to approximately 27 wt% of α-Fe_2_O_3_ relative to the total Fe based content, while the pyrolysis under N_2_ atmosphere favors the reduction of α-Fe_2_O_3_ (found only at 3 wt%) to lower states, mostly to a mixture of the intermediary γ-Fe_2_O_3_ with Fe_3_O_4_. 

**Figure 3:**
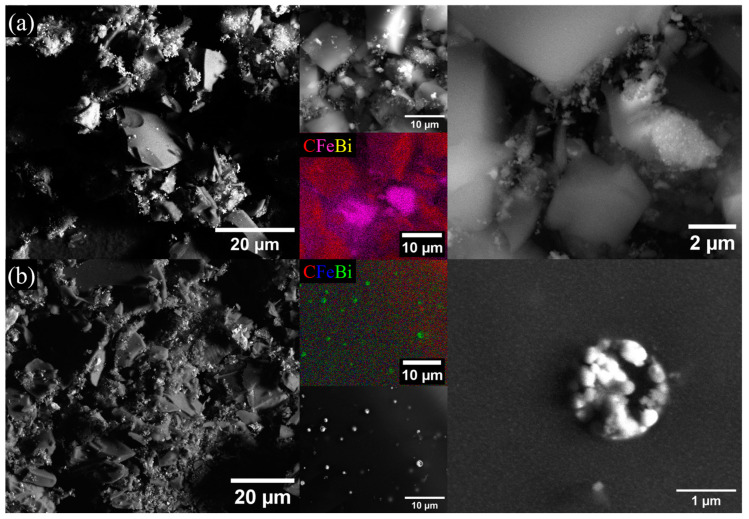
SEM micrographs and elemental maps for XG samples treated under (**a**) Ar atmosphere and (**b**) N_2_ atmosphere, revealing the composite-specific features and phase distribution at micrometer scales.

The SEM analysis performed after fine grinding the nanocomposites treated under Ar and N_2_ atmospheres showed that the previously observed features (i.e., fractal surface, fine nanoparticle dispersion in the carbon support) are intermixed, achieving the complex metal/oxide/carbon powders presented in Figure 3. At low magnifications, using elemental maps, Fe is well evidenced with free standing features between carbon particles (purple values in the first EDX map), while Bi could only be revealed using EDX analysis. Also, some carbon grains exhibited well-dispersed metal oxide nanoparticles, and some interesting hierarchic microspheres were found close to their surface having an elemental composition rich in Bi (green values in the second EDX map). It is considered that due to the low melting point of Bi, during pyrolysis, highly mobile melts will evolve through the pores and cracks of the xerogels and give rise to micro-structures similar to the ones observed in Figure 3 through coalescence and Ostwald ripening mechanisms. This also explains why the concentration of Bi is low in these particular systems and why clear signals could not be observed in the XRD analysis.

**Figure 4:**
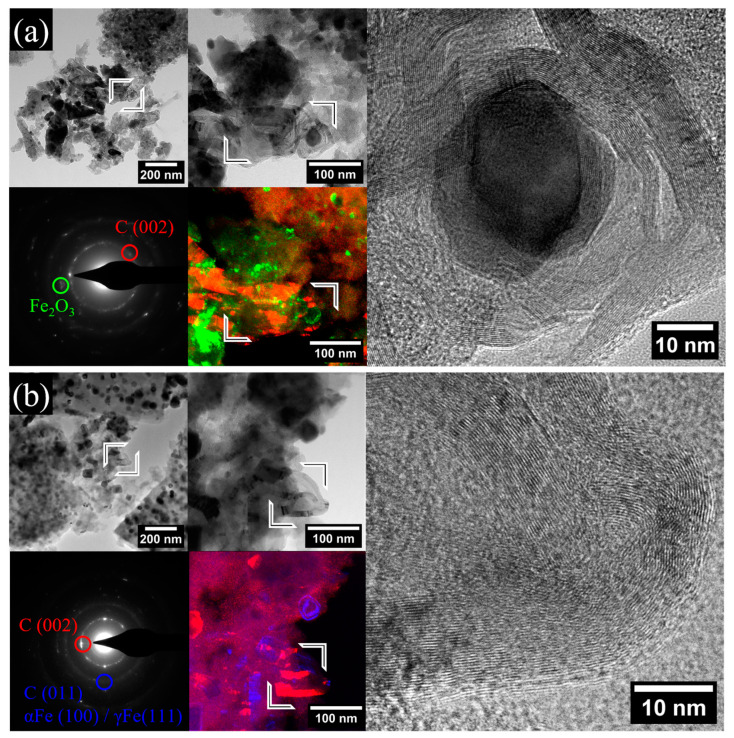
TEM micrographs obtained in bright field, dark field, and HRTEM modes for (**a**) XGAr and (**b**) XGN nanocomposites. The dark-field images are stacked images obtained using electrons scattered from carbon (002)-specific regions (red values), adjacent Fe_2_O_3_-specific regions (green), and carbon (110)/metallic Fe-specific regions (blue values) from the indicated SAED patterns.

The nanosized features of the investigated nanocomposites are emphasized in the TEM micrographs presented in Figure 4. Metal/oxide systems are observed in various shapes and sizes, such as nanoparticles with sizes between 5 and 60 nm, dispersed in the carbon matrix, which are specific to co-synthesized meso-aggregates, microcrystals, and tear drop-shaped particles with sizes between 5 and 300 nm that mostly occur due to the Fe impregnation protocol and high-temperature conditions. Some differences regarding the size of carbon-embedded nanoparticles were noted between the Ar and N_2_ treated samples, as larger values were observed for the latter one. This is reflected by the mean diameter (<D_TEM_> in Table 1) values of 17 and 26 nm for XGAr and XGN, respectively. Also, the TEM investigations indicate the activation of the heterogeneous graphitization mechanism during which complex graphitic structures similar to nanofibers, tubes and shells are observed. As shown in Figure 4, the improved crystallinity of the carbon nanostructures is evidenced by the appearance of strong (002) and (110) diffracting rings observed in the SAED patterns, and the appearance of (002) stacked planes in HRTEM mode with an average interplanar spacing of 3.5 Å, specific to carbon nanotubes and nanofibers.

The crystalline contrast between the components of the nanocomposite can be exploited in the dark-field imaging mode. Thus, stacked dark-field images were obtained by selecting the Bragg scattered electrons from different regions from the SAED patterns, for example from the carbon-(002) plane (presented with red values in the dark-field composite images), reflections adjacent to the main regions for Fe_2_O_3_ (presented with green values in the composite dark-field (CDF) stacks) and the regions specific to the αFe-(110), γFe-(111) and carbon-(110) planes (presented with blue values in the CDF stacks). Hence, one can clearly distinguish the carbon ribbons and the basic structural units constituting the amorphous carbon matrix. These units forming the amorphous support were observed to have sizes below 3 nm. The graphitized carbon ribbons show a continuous stacked structure with thicknesses between 5 and 40 nm and lengths beyond 200 nm. Such features were not observed in the previous co-synthesized samples and are related to the high yield in metallic and iron carbides [12].

Due to its sensitivity to various iron oxide and carbon structures, micro-Raman spectroscopy was used to probe nanocomposites. The most significant results are discussed in terms of the signal variations observed at small wavenumber values (100–800 cm^−1^) specific to iron oxide sites and the characteristic D-G signals (1000–1620 cm^−1^) gathered from the carbonaceous surfaces exposed after grinding. 

**Figure 5:**
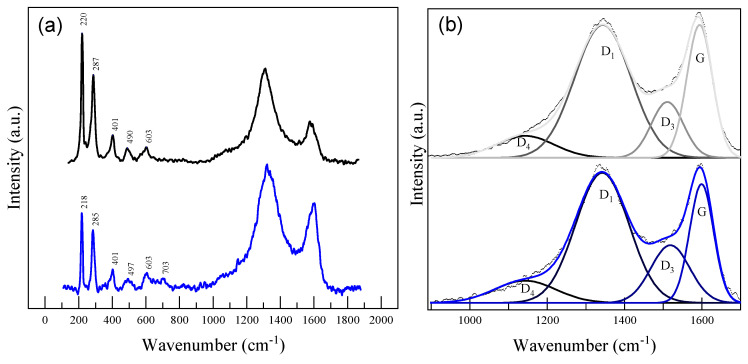
Raman spectra of the XGAr (black) and XGN (blue) nanocomposites recorded from (**a**) Fe oxide-rich regions and (**b**) carbonaceous grains. Deconvoluted signals D and G are also presented (see text for details).

The powdered samples were analyzed using small laser power outputs (between 2 and 10 mW) to minimize any laser beam effects [20]. The representative spectra are shown in Figure 5. For the Ar-treated sample, the spectra acquired from the sites having iron oxide-specific hues exhibited bands at 220, 287, 401, 490, and 603 cm^−1^, with a higher intensity relative to the carbon-specific signals found between 900 and 1800 cm^−1^. The bands’ assignments are presented in Table 2. Even though the peak positions are characteristic to hematite, the relative intensities of the bands observed at 287 and 219 cm^−1^ were found close to 0.68, which is not specific to this phase. Similar spectra were obtained by Shebanova et al. during in situ heating of magnetite powders when exceeding a laser power of 18 mW [20]. The obtained signals were attributed to a mixture of metastable maghemite and hematite phases. As expected, the XGN sample revealed similar signals, but with smaller intensities. Other weak signals around 703 cm^−1^ were also detected that may indicate an increase in the crystallinity of the maghemite phase. The results are in agreement with the XRD findings.

With regard to the Raman-derived information for the carbon component of the nanocomposites, the signals around 1350 cm^−1^ represent the defect-mediated double resonance D band, while the signal around 1590 cm^−1^ stands for the G band associated with the in-plane stretching vibrations of sp^2^ atoms found in graphitic structures [21]. The G band is generally associated with a graphitic-like structure, while the D band is linked with the level of disorder. Therefore, several features, such as the FWHM values for the two bands or the ratio between the D band and G band intensities (I_D_/I_G_), are largely used to quantify the disorder in carbonaceous materials [22]. As observed in the recorded spectra, within the oxygen-rich sites where iron oxides are present, the D band was more prominent and indicates a more defective structure of carbon. However, the carbonaceous grains that are to be exposed after grinding represent the dominant carbon fraction. The D and G bands specific to the exposed grains indicate different contributions. Due to the amorphous nature and small in-plane crystallite dimension of the carbon xerogels, the D and G bands significantly overlap. For such species, reports indicate that other carbon-related signals need to be taken into account, and a multi-peak fit is required to extract the parameters related to the carbon bands. Starting from the analysis performed by Alegre et al. on similar materials, a four-peak fit is considered [22], and the fit results are summarized in Table 2. The findings indicate similar contributions of each band for both Ar- and N_2_-treated composites; nevertheless, the N_2_ sample exhibited smaller I_D_/I_G_ and smaller FWHM values for the D and G bands, which suggests the existence of a higher crystallinity of at least one carbon phase present in the carbon grains.

**Figure 6:**
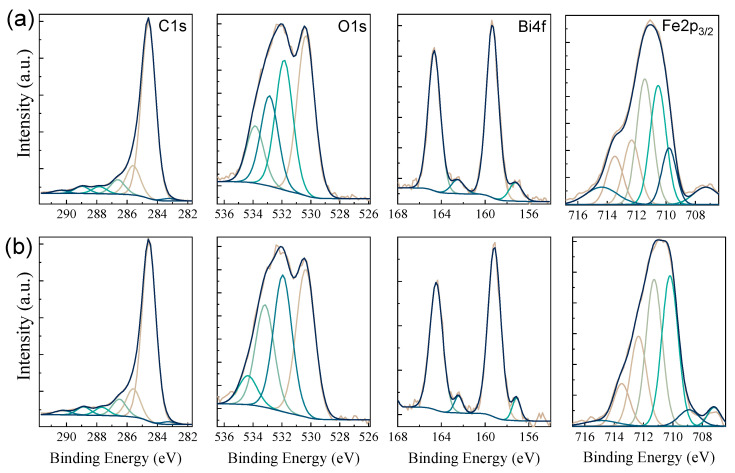
XPS spectra showing the fitted C1s, O1s, Bi4f, and Fe2p regions for (**a**) XGAr and (**b**) XGN nanocomposites.

The high resolution C1s, O1s, Bi4f, and Fe2p XPS spectra recorded for XGAr and XGN samples are illustrated in Figure 6. The elemental composition of the samples surface provided by the XPS wide-scan spectra indicates small variations, as reported in Table 1. For the composite treated under N_2_, a slightly increase in the Fe contribution (1.7 at% vs. 1.2 at%) is observed. The deconvolution results performed on the main peaks are also summarized in Table 3.

The asymmetric profile of the C1s peak centered at 284.6 eV is described using seven contributions, which are ascribed to sp^2^ (284.5 eV) and sp^3^ (285.6 eV) species found in the carbon matrix and defect-specific species, such as C-O epoxy/alcoxy/ethers groups (286.5 eV), C=O carbonyls (287.7 eV), O-C=O carboxyl groups (288.9 eV). Also, as specific to graphitic structures, two signals associated with the π-π* shake-up satellite (290.3 eV) and carbon edge defects (283.3 eV) were observed. Complementary to the XRD, TEM, and Raman findings, the deconvolution results support the fact that more reactive conditions are obtained in the N_2_ atmosphere that will tend to affect both the carbon supports and the reduction state of the embedded nanoparticles.

The O1s spectral region can be viewed as a superposition of surface species contained in carbon groups and species found in oxides. For the deconvolution of the O1s region, the best results were obtained using four components. For the oxide-related region, the bands were ascribed to lattice oxygen in iron and bismuth oxides (Fe-O and Bi-O ~530.3 eV) and adsorbed hydroxyls on oxide surfaces (~531.8 eV). The components for the carbon matrix were associated with C-O and C=O-O groups (~533.8 eV and ~534.3 eV, respectively). It is observed that, under N_2_ atmosphere, the hydroxyl-like contributions are larger relative to the lattice oxygen signal, which may reveal a more hydrophilic nature of the XGN possibly due to higher hematite to maghemite conversion yields [23].

After the pyrolysis step, the presence of metallic Bi particles was expected, but only minute contributions were observed from the XRD analysis. Due to its high surface sensitivity and broad spatial analysis, the Bi component was better evidenced through XPS analysis. The Bi 4f region is characterized by the two Bi 4f_5/2_ and Bi 4f_7/2_ peaks separated by a 5.3 eV gap. The peaks centered at 164.6 and 159.3 eV were assigned to Bi^3+^ species in Bi_2_O_3_. A second Bi_4f_ doublet was observed at 157.2 and 162.5 eV, confirming the presence of Bi in a metallic state. The small Bi/Bi_2_O_3_ ratio is explained by the formation of Bi_2_O_3_ shells around the Bi particles that takes place due to the oxygen impurities during pyrolysis and exposure to the environment [10].

Similarly, Fe2p-specific regions indicate a dominant contribution by iron oxide species and small traces of Fe^0^ species. To further distinguish between different chemical states of Fe oxide, the Fe2p_3/2_ region was fitted based on the multiplet splitting procedure as elsewhere described [24]. The multiplet signals consist of four bands centered around 709.0, 710.3, 711.3, 712.3, and 713.5 eV to which a low contribution due to metallic states was added at 707.2 eV and a broad peak at 714.4 eV correlated with surface structures and shake-up satellite [24]. The high binding energy (B.E.) asymmetry is lower for XGN, which in turn exhibits a slight increase in the contributions to the low B.E. The peaks are wider, and the positions are shifted towards lower B.E. so that a 708.9 eV band is distinguished. Such features are associated with increased yields of Fe^2+^ states [24,25]. This suggests that even though Fe^3+^ sites, mostly found in γ-Fe_2_O_3_ for both XGAr and XGN, will be dominant, N_2_ treatment activates an incipient transition towards Fe_3_O_4_/FeO systems. Interestingly, as observed from XPS and XRD investigations, Fe^0^ states are reached with higher yields than intermediary Fe^2+^ stable states, probably due to higher stability and oxygen shields owed to the carbon encapsulation.

### 2.2. Electrochemical Performances

#### 2.2.1. Electrochemical Characterization of XGAr- or XGN-Based Modified Electrodes

**Figure 7:**
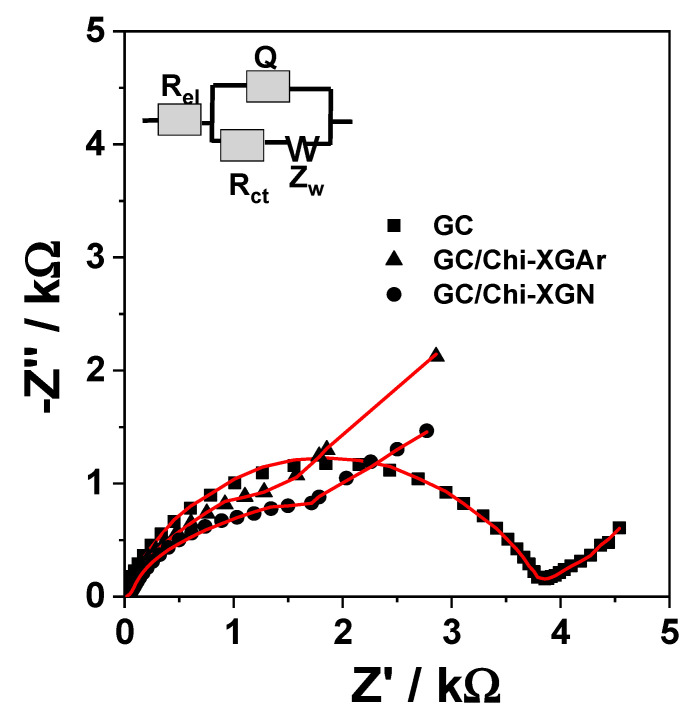
EIS spectra of GCE and GCE/Chi-XGAr or GCE/Chi-XGN. Experimental conditions: supporting electrolyte, 0.1 M acetate buffer (pH 4.5) containing 1 mM [Fe(CN)6]^3−/4−^; applied potential, 0.2 V vs. Ag/AgCl,KClsat; frequency interval, 0.1–10^4^ Hz.

The interface properties of GC/Chi-XGAr, GC/Chi-XGN, and GC electrodes were established with electrochemical impedance spectroscopy (EIS) using [Fe(CN)6]^3−/4−^ as the electrochemical probe (Figure 7). The experimental results were fitted with a modified Randless equivalent circuit [10,11] to obtain the electric parameters (Table 4). The equivalent circuit includes an uncompensated resistance of the electrolyte solution (R_el_) coupled in series with a parallel arrangement of the interface capacitance (Q) and faradaic impedance. The interface capacitance is modelled as a constant phase element (CPE) combined with a double layer capacitance (C). The faradaic impedance represents a corroborated effect of a charge transfer resistance (R_ct_) and a mass transfer resistance (W).

Comparing the R_ct_ value obtained for bare GC electrode and XGAr- or XGN-based modified electrodes, it is clear that the presence of the XG nanocomposite in the electrode configuration led to a substantial decrease in the charge transfer resistance (R_ct_). Moreover, the R_ct_ value is higher for the electrode prepared with XGAr in comparison with the value achieved when the electrode contained XGN. The small difference in the R_ct_ value obtained for XGN and XGAr can be provided by different amounts of metal/metal oxide nanoparticles present in the composite matrix (Table 1 and Table 3). Thus, XGN with more Bi nanoparticles presents lower R_ct_. Moreover, the corroborated effect of the nanocomposite matrix properties (nanoparticle shape, size, crystalline phase, etc.) (Table 1) strongly influence the double-layer capacitance (C) (Table 4), reflected by the following sequence: GC ˂ GC/Chi-XGN ˂ GC/Chi- XGAr.

Interestingly, in the region corresponding to lower frequencies and the domain attributed to diffusion-limited processes, a similar behaviour for GC/Chi-XGAr and GC/Chi-XGN was observed (Figure 7).

#### 2.2.2. Amperometric Detection of H_2_O_2_

The functionality of the investigated XGAr or XGN nanocomposite as electrode materials for H_2_O_2_ detection is evidenced by the response of the modified GC/Chit-XGAr or GC/Chit-XGN electrodes in amperometric measurements. From cyclic voltammograms recorded using XGAr or XGN nanocomposite-modified electrodes in phosphate buffer solution (results not shown), the applied potential value for amperometric measurements was achieved (i.e., −0.3 V vs. Ag|AgCl,KCl_sat_). H_2_O_2_ reduction occurs, as proven before [9,26], through an electrocatalytic process that involves two sequential steps: oxidation of Fe^+2^ ions by H_2_O_2_, according to a Fenton-type mechanism, and reduction of the chemically produced Fe^+3^ cations, thus obtaining the catalyst regeneration. The corresponding reactions are noted as follows:

$$Fe^{2+}+H_{2}O_{2}\to Fe^{3+}+HO\cdot +OH^{-}\;Fe^{3+}+e^{-}\to Fe^{2+}$$

**Figure 8:**
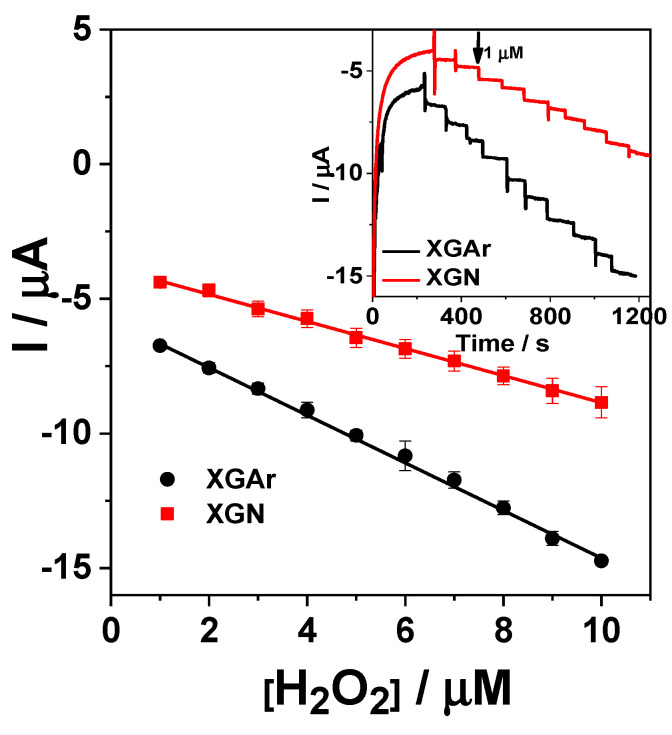
Amperometric calibration curve corresponding to I vs. time dependence (Inset) recorded at GC/Chi-XGN or GC/Chi-XGAr upon the successive addition of 1 µM H_2_O_2_. Experimental conditions: rotating speed, 400 rpm; supporting electrolyte, 0.1 phosphate buffer M (pH 7); applied potential, −0.3 V vs. Ag/AgCl,KCl_sat_.

Using an amperometric technique, after successive additions of 5 µM H_2_O_2_ to the electrolyte solution_,_ typical current time response curves were obtained for both GC/Chi-XG-modified electrodes (Figure 8 inset). The signal stabilization at GC/Chi-XG was achieved fast (less than 8 s), demonstrating the GC/Chi-XG competitiveness. Based on the recorded results, the amperometric calibration curves (Figure 8) were used to prove the GC/Chi-XG performances for H_2_O_2_ reduction. The corresponding linear regression was described by the following equations: I/A = (−3.912 ± 0.061)·10^−6^ + (−0.508 ± 0.010)·[H_2_O_2_/M] (R^2^ = 0.99585, and N = 10) for GC/Chi–XGN and I/A = (−5.773 ± 0.044)·10^−6^ + (−0.905 ± 0.007) [H_2_O_2_/M] (R^2^ = 0.99931, and N = 10) for GC/Chi–XGAr. The calculated electroanalytical parameters are shown in Table 5. Comparing the results obtained in the recorded linear range from 5 to 50 µM for GC/Chi-XG, a higher sensitivity and a lower detection limit (for a signal-to-noise ratio of 3) were observed for the XGAr-based modified electrode in comparison with the competing one. A possible explanation for the obtained results can be the presence of α-Fe_2_O_3_ in different amounts in the compared samples XGN or XGAr (Table 1)_._ Consistent with the results obtained by C.-Y. Lin et al., who showed that among iron oxide nanorods (β-FeOOH, α-Fe_2_O_3_, γ-Fe_2_O_3_), only α-Fe_2_O_3_ was found to be active in the phosphate buffer [27], our work on the XGAr and XGN nanocomposite electrode materials evidenced that α-Fe_2_O_3_ presence or absence can influence the electroanalytical results.

A comparison with other already published results (Table 5) proved that the developed GC/Chi-XGN or GC/Chi-XGAr electrodes are competitive based on the electroanalytical parameters for H_2_O_2_ detection, which are superior or comparable to those of the previously reported ones. Detection limits four and eight times lower for GC/Chi-XGAr and GC/Chi-XGN, respectively, were obtained comparatively with that reported by Heydaryan et al. [30] who used expensive materials, such as Au, Ag, and RGO, for electrode preparation. There seems to be a compromise in the linear range and detection limit. Thus, relinquishing to a large linear range leads to a low detection limit. Based on the previous remark, a detection limit almost three orders of magnitude lower and a linear range three orders of magnitude smaller were obtained for the Fe_3_O_4_ nanoparticle/r-GO nanosheet-modified glassy carbon (Fe_3_O_4_/r-GO/GC) electrode [29].

#### 2.2.3. Voltammetric Detection of Pb^2+^

**Figure 9:**
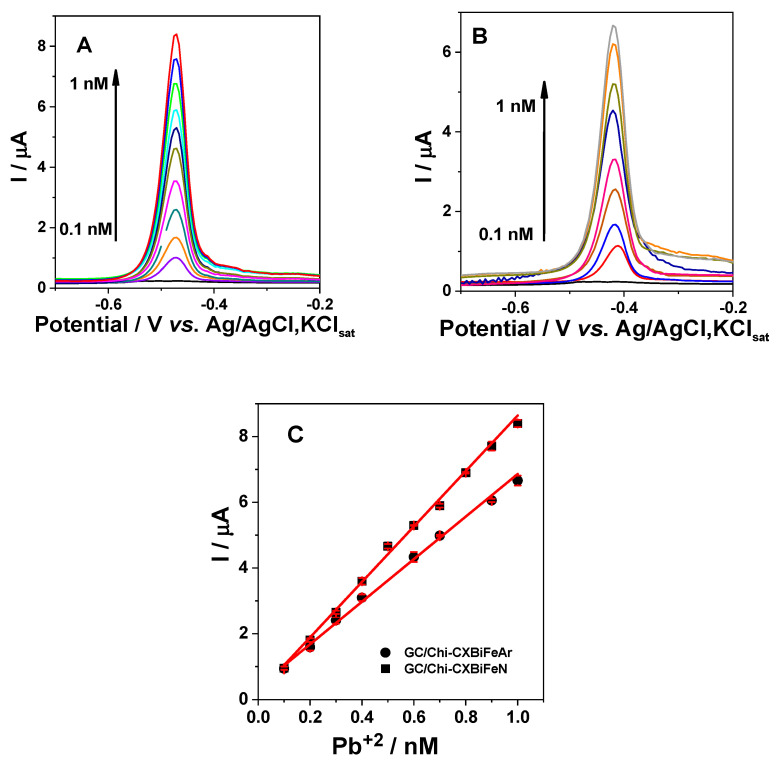
SWASVs recorded at GC/Chi-XGN (**A**) and GC/Chi-XGAr (**B**) electrodes in the presence and absence of Pb^2+^ and the corresponding calibration curves (**C**). Experimental conditions: supporting electrolyte, 0.1 M acetate buffer (pH 4.5); deposition potential, −1.4 V vs. Ag/AgCl,KCl_sat_; deposition time, 180 s; frequency, 10 Hz; amplitude, 25 mV; starting dissolution potential, −1.2 V vs. Ag/AgCl,KCl_sat_.

The possible use of the nanocomposite as electrode material for heavy metal detection applications is revealed using the SWV technique as further presented. The recorded voltammograms in the presence of Pb^2+^ (0.1–1 nM) in 0.1 M acetate buffer (pH 4.5) at GC/Chi-XGAr and GC/Chi-XGN electrodes show a clear anodic peak, corresponding to the oxidation of Pb previously reduced on the surface of the electrode during the preconcentration step (Figure 9A,B). 

The experimental results were used to draw the calibration curves (Figure 9C), and the calculated regression line was described by the following equations: I/A = (1.97 ± 0.93)·10^−7^ + (8.44 ± 0.15)·10^3^[Pb^2+^/M] (R^2^ = 0.99707, and N = 10) for GC/Chi–XGN and I/A = (0.38 ± 0.10)·10^−6^ + (6.47 ± 0.15)·10^3^[Pb^2+^/M] (R^2^ = 0.99606, and N = 8) for GC/Chi– XGAr. These equations allow the estimation of the electroanalytical parameters for Pb^2+^ detection. GC, glassy carbon; Chi, chitosan; CX, carbon xerogel; CA, carbon aerogel; G, graphene; MWCNTs, multiwall carbon nanotubes; rGO, reduced graphene oxide.

The anodic peak potential values for Pb oxidation at GC/Chi-XG electrodes present slight differences (Table 6) that can be related to the surface hydrophobicity of the nanocomposite material [10]. Furthermore, the obtained sensitivity value for GC/Chi-XGN was ~25% higher than that obtained for GC/Chi-XGAr, and the detection limit value (estimated for a signal to noise ratio of 3) for Pb^2+^ recognition was 30% lower than that obtained for GC/Chi-XGAr (see Table 6). Thus, the higher sensitivity and the lower detection limit obtained at the XGN-based modified electrode can be correlated with the higher amount of Bi (from XPS measurements) and the composite high conductivity and low capacity in comparison with those obtained for the XGAr nanocomposite-based electrode (Table 4 and Table 6).

Moreover, the obtained electroanalytical parameters for Pb^2+^ detection at GC/Chi-XGAr and GC/Chi-XGN can be also due to the synergy of the individual or cumulative effects of the crystalline phase, size, and distribution of the Bi/Fe nanoparticles in XG matrix as well as the proportion among them (Table 1).

The affinity of heavy metals (Pb^2+^) for environmentally friendly Bi (reduced) is the main factor that influences the performances for heavy metal detection. Thus, the extremely lethal Hg-based devices, which have long been proven to be proper for the sensitive detection of heavy metals was substituted. In this context, the generation of Bi-based material electrodes following a similar approach to that previously used with Hg [1] has been of widespread application in research. Moreover, the current intensities of the different heavy metals were proportional to the amount of Bi in the form of nanoparticles present in the electrode material [1,10]. Therefore the Bi active surface area directly depends on the amount of Bi in the XG materials, but it is not directly proportional due to the fact that with the Bi concentration increasing not only the number of Bi nanoparticles increase by preserving its particle size distribution, but also lead to a larger particle sizes. The obtained electroanalytical parameters recommend both XGN- and XGAr-modified electrodes as competitive for Pb^2+^ detection, comparable with already published results (Table 6). Interestingly, in comparison with our previous works [12], a linear range two orders of magnitude larger, a sensitivity two orders of magnitude smaller, and a limit of detection two orders of magnitude higher were obtained. Even so, the obtained values for the limit of detection for Pb^2+^ are far below the standard level recommended by the WHO and EU for drinking water [4,5].

#### 2.2.4. The Operational Stability (Reusability) of GC/Chi-XGAr and GC/Chi-XGN Electrodes

The composite-modified electrode surface stability represents an important characteristic of the modified electrodes used as analytical sensors and is reflected in the electrodes’ reusability (operational stability) results. This was estimated for both investigated XGAr and XGN electrode composite materials by measuring the relative standard deviations of the recorded signal for studied analytes (Pb^2+^ and H_2_O_2_, respectively) at the same modified electrode for 3 successive measurements under the same experimental conditions (Table 7). It can be stated that the reproducibility of current signal was better for measurements performed by SWV than that obtained by amperometric measurements and can be reflected by the technique specificity. Furthermore, in the case of Pb2+ detection, the reproducibility of peak current intensities was better than that observed for peak potential for both GC/Chi-XGAr- and GC/Chi-XGN-modified electrodes, namely 0.5%.

**Figure 10:**
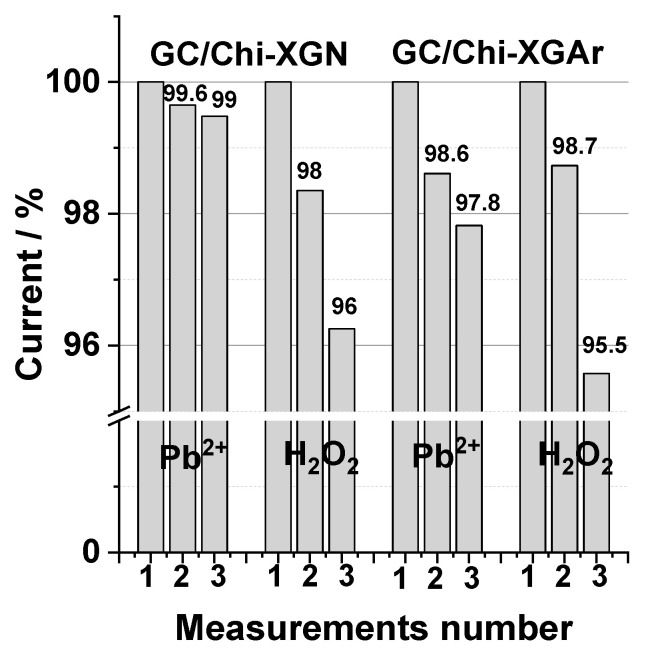
Operational stability of GC/Chi-XGN and GC/Chi-XGAr for three successive recorded measurements for 0.7 nM Pb^2+^ and 15 µM H_2_O_2_ detection. For experimental conditions, see Figure 8 and Figure 9.

Moreover, the results show that for the composite modified electrodes used 3 times for Pb^2+^ detection or H_2_O_2_ detection (Figure 10), there is a slow decrease in the sensors response (~5% for both XGAr and XGN from the initial response). Based on these results, it can be concluded that stable and efficient sensors can be achieved using a simple modification technique (drop casting) of the GC electrode surface with new composite materials XGAr or XGN.

#### 2.2.5. Time Stability

The time stability of the obtained modified electrodes (GC/Chi-XGN and GC/Chi-XGAr) was estimated by performing SWV voltammograms and amperometry immediately after preparation and six months later for three electrodes in the presence of 0.7 nM Pb^2+^ or 15 µM H_2_O_2_. The experimental conditions are those presented in Figure 8 and Figure 9. During this period, the electrodes were kept in ambient conditions (room temperature, room humidity). The obtained results achieved as the average of peak currents for both analytes showed a slight decrease in time: for Pb2+, 4.03% at GC/Chi-XGN and 3.45% at GC/Chi-XGAr; for H_2_O_2_, 3.45% at GC/Chi-XGN and 4.62% at GC/Chi-XGAr.

It should be mentioned that after the prepared suspensions were kept in ambient conditions for one year, the obtained peak intensity recorded at GC/Chi-XGN and GC/Chi-XGAr under the previous experimental conditions in the presence of 0.7 nM Pb^2+^ resulted in a decreased of about 5% for both prepared electrodes.

Therefore, the obtained results proved that there is no surface fouling or degradation at the developed nanocomposite-based modified electrodes and that these electrodes exhibit good repeatability, reproducibility, and time stability for the determination of Pb^2+^ or H_2_O_2_.

#### 2.2.6. Real Sample Analysis

To illustrate the application of the proposed modified electrodes (GC/Chi-XGN and GC/Chi-XGAr) in real samples analysis, the electrodes were employed to detect Pb^2+^ in drilling water (Jibou, District of Salaj, Romania). The concentration of Pb^2+^ found with GC/Chi-XGN was (1.96 ± 0.05)·10^−10^ M (with relative error (%) of +4.39 and RSD (%) 2.5) and with GC/Chi-XGAr was (1.99 ± 0.09)·10^−10^ M (with relative error (%) of +2.92 and RSD (%) 4.66). The results of the electrochemical experiments realized by SWV and standard addition methods (in the same conditions as calibration curve) are in a good agreement with those obtained by atomic absorption spectroscopy (AAS), a standardized method (2.05·10^−10^ M M), which confirmed the accuracy and reliability of the approach.

## 3. Conclusions

Monolithic carbon xerogels obtained by co-synthesis with bismuth and iron precursors were further iron enriched by following an additional impregnation route. Pyrolysis treatment at 1050 °C was performed under Ar and N_2_ to evaluate any changes in the material parameters in terms of the metal oxide reduction yield and the occurrence of any C-Fe interactions. SEM/EDX and XPS analyses confirmed that the new composites contain higher content of iron phases located both at the surface of the monolithic samples and in the inner layers in comparison with previously reported CBiFe xerogel nanocomposites. Bismuth was detected in low concentrations, both in reduced and oxidized states, while four types of Fe-related structures were identified: hematite (α-Fe_2_O_3_), maghemite (γ-Fe_2_O_3_), ferrite (α-Fe), and austenite (γ-Fe). The difference between the two pyrolysis conditions was revealed using XRD, Raman spectroscopy, and XPS investigations, where a higher presence of reduced Fe states was evidenced for the sample treated under N_2_ due to its more reactive nature. Moreover, it was found that the pyrolysis under Ar atmosphere favors an increase in the α-Fe_2_O_3_ amount and H_2_O_2_ detection performance (with a sensitivity of 0.9 A/M and limit of detection of 0.17 μM), while the pyrolysis under N_2_ atmosphere favors an increase in the metallic Bi amount and Pb^2+^ detection performance (with a sensitivity of 8.44·10^3^ A/M and limit of detection of 33.05 pM). The acquired results were correlated with those obtained by morphological, structural, and surface investigations.

## 4. Materials and Methods

### 4.1. Reagents

All reagents were of analytical grade and were used without any further purification. The following agents were bought from Sigma Aldrich (St. Louis, MO, USA) unless otherwise specified: resorcinol (m-C_6_H_4_(OH)_2_, 99%), formaldehyde solution (37 wt% in H_2_O, stabilized with methanol, Chem-Lab, West-Vlaanderen, Belgium), bismuth (III) nitrate pentahydrate [Bi(NO_3_)_3_·5H_2_O, 98%, Alfa Aesar (Haverhill, MA, USA)], acetic acid (CH_3_COOH, 99.7%), anhydrous iron (II) acetate (Fe(OOCCH_3_)_2_, minimum Fe content 29.5%), acetic acid (CH_3_COOH, 99%), ammonium hydroxide water solution (NH_3_aq., 10 wt%), and glycerol formal (47–67% 5-hydroxy-1,3-dioxane, 33–53% 4-hydroxymethyl-1,3-dioxolane). Bidistilled water was used for solution preparation.

### 4.2. Synthesis of Xerogel Ternary Composites

Xerogel composites were prepared using a modified resorcinol formaldehyde sol–gel synthesis method previously described [12]. Here, 1.2 g Bi(NO_3_)_3_·5H_2_O was dissolved under of stirring in 10 mL glycerol formal for about 2 h. Then, resorcinol (R) as 2 g mass and formaldehyde (F) as 37% solution were added to a molar ratio R/F = 0.5. A volume of 4 mL solution of NH_4_OH (10% wt) and 12 mL of acetic acid were next added. The iron salt as 0.06 g Fe(OOCCH_3_)_2_ was dissolved into the obtained mixture (i.e., the co-synthesis pathway for the first iron insertion). 

The solution was placed in sealed glass vessels and held for 3 days at 60 °C. The obtained wet gel was then rinsed with ethanol and held in acetic acid for one day for washing. After a second step of several rinses with ethanol, the gel was placed for one day in 0.1 M Fe(OOCCH_3_)_2_ ethanolic solution (i.e., the impregnation pathway for the second iron insertion) and dried under ambient conditions for more than 5 days until no change of the mass was noticed. The obtained organic xerogel impregnated with well-dispersed Bi and Fe cations was pyrolyzed at 1050 °C for 1 h using a heat rate of 3 °C/min and under Ar or N_2_ atmospheres. Two types of carbon xerogels (XGAr and XGN) embedded with Bi and Fe nanoparticles were obtained. 

### 4.3. Characterization Methods

X-ray diffraction (XRD) measurements were performed on a Shimadzu 6000 diffractometer using Cu-Kα radiation (λ = 1.5406 Å) equipped with a graphite monochromator. The diffractograms were recorded between 2θ = [15, 70°] with a step size of 0.05°, 0.5°/min. For the phase identification, the AMCSD database [35] was employed. A quantitative phase analysis of the Fe-based phases was performed using the Maud software [36] and the COD database [37].

A FEI Quanta 3D FEG dual beam in high vacuum mode using an EDT (Everhart Thornley Detector) scanning electron microscope (equipped with an ApolloX SDD Energy Dispersive X-ray (EDX) detector) was used for investigating the surface and elemental composition of the pyrolyzed monoliths.

Transmission electron microscopy (TEM) was performed on the grinded samples dispersed in water. The micrographs acquired in bright-field (BF), dark-field (DF), and high-resolution (HRTEM) modes together with selective area electron diffraction patterns (SAED) were obtained using a FEI Tecnai G2 F20 TEM operating at an accelerating voltage of 200 kV.

Raman spectra were recorded with a Renishaw inVia Reflex Raman microscope equipped with a RenCam CCD detector. The 532 nm laser was used as excitation source, and the spectra were collected using a 0.9 NA objective of 100× magnification. Typical integration times were 10–20 s, and the laser power was 2–10 mW. The Raman spectra were recorded with a resolution of 2 cm^−1^.

XPS spectra were recorded with a SPECS PHOIBOS 150 MCD system using a monochromatic Al-Kα source (1486.6 eV), a hemispherical analyzer multichannel detector, and a charge neutralization device. Samples were fixed on double-sided carbon tape, and care was taken to ensure that the sample particles covered the tape. Experiments were performed by operating the X-ray source with a power of 200 W, while the pressure in the analysis chamber was in the range of 10^−9^ to 10^−10^ mbar. The binding energy scale was charged referenced to the C1s at 284.6 eV. Elemental composition was determined from survey spectra acquired at a pass energy of 60 eV. High-resolution spectra of the C1s, O1s, Fe2p and Bi4f regions were obtained using analyzer pass energy of 20 eV. Analysis of the data was carried out with Casa XPS software 2.3.18.

### 4.4. Preparation of the GC/Chi-XGAr_r_ and GC/Chi-XGN Electrodes

The glassy carbon electrode (GCE, 3 mm diameter) was polished before each experiment with 1 μm and 0.05 μm alumina powder (Stuers, Copenhagen, Denmark), separately, and rinsed carefully with bidistilled water between each polishing step. Then, to remove the alumina particles and other possible contaminants, the GCE surface was successively washed with acetone and bidistilled water in ultrasonic bath and dried in air. The XG nanocomposite-modified electrode was prepared by casting 5 μL of XG suspension in chitosan on the GCE surface and dried under a beaker for 2 h at room temperature. For the preparation of XG suspension in chitosan, a solution of 10 mg chitosan (Chi) in 10 mL of 0.1 M acetic acid was prepared. Then, by adding 1 g/L XG powder and sonication for 2 h, the suspension was obtained.

### 4.5. Electrochemical Measurements

The electrochemical measurements were conducted using a PC controlled electrochemical analyzer (AUTOLAB PGSTAT302N EcoChemie, Utrecht, The Netherlands) with a three electrode configuration at room temperature. The modified GC/Chi-XGAr or GC/Chi-XGN serves as a working electrode, Ag/AgCl in saturated KCl solution works as a reference electrode, and a Pt wire was the counter electrode.

The electron transfer abilities of the GCE and XGAr or XGN nanocomposite-modified electrodes were investigated with electrochemical impedance spectroscopy (EIS) using 0.1 M phosphate buffer containing 5 mM [Fe(CN)_6_]^3−/4−^ in a frequency range from 10^4^ Hz to 10^−1^ Hz. The ZSimpWin 3.21 software was used to fit the experimental results with a modified Randless equivalent circuit [9].

The electroanalytical experiments were performed using square wave voltammetry (SWV) and amperometric techniques. The heavy metal (i.e., Pb^2+^) detection was carried out in 0.1 M acetate buffer (pH 4.5) by SWV. Thus, after Pb^2+^ deposition at −1.4 V vs. Ag/AgCl,KCl_sat_ for 180 s, under constant stirring at 400 rpm, and 10 s of equilibration after stirring was ceased, the voltametric scan was performed. The starting dissolution potential was −1.2 V vs. Ag/AgCl,KClsat, the frequency was 10 Hz, and the amplitude was 25 mV. The electroanalytical behaviour of GC/Chi-XGAr or GC/Chi-XGN was studied for the detection of 0.1–1 nM Pb^2+^.

The amperometric experiments were used for hydrogen peroxide detection (1–10 µM H_2_O_2_), and the investigations were carried out in 0.1 M phosphate buffer (pH 7) at an applied potential of −0.3 V vs. Ag|AgCl,KClsat. 

All experiments were carried out in aerated solutions.

## Figures and Tables

**Table 1:** Obtained morphological and structural parameters of the XGAr and XGN nanocomposites.

Sample	Fe Crystalline Phase Analysis (wt%)				I_D_/I_G_	<D_TEM_> (nm)	C/O/Bi/Fe (at%)
	α-Fe_2_O_3_	γ-Fe_2_O_3_/Fe_3_O_4_	α-Fe	γ-Fe			
XGAr	27	6/13	17	37	1.00	17	88/10.5/0.3/1.2
XGN	3	2.2/44	18	33	1.09	26	87.8/10.3/0.2/1.7

**Table 2:** Main Raman signals observed (1) in the low wavenumber region specific to the Fe oxide vibrations and their assignment according to the literature (bold values indicate the most intense band) and (2) in the 1st order D-G carbon vibrations region treated as 4 superposed signals.

Iron Oxide						
Sample				Reference Signals [18]		
XGAr		XGN		α-Fe_2_O_3_	γ-Fe_2_O_3_	Fe_3_O_4_
220		218		220 A_1g_		200 T_2g_
287		285		<300 E_g_(3)		306 E_g_
401		401		410	400	
490		497				500 T_2g_(2)
603		603		600 E_g_(5)	600	
		703			700	665 A_1g_
**Carbon**						
**Sample**	**Spectral Band**	**Peak Type**	**Area Fit** **(%)**	**Position** **(cm^−1^)**	**Max Height** **(Normalized)**	**FWHM** **(cm^−1^)**
XGAr	D_4_	Gaussian	9.04	1142	15.12	175
	D_1_		53.56	1342	91.53	171
	D_3_		12.81	1511	38.43	97
	G		24.59	1594	91.51	78
XGN	D_4_	Gaussian	10.18	1143	15.27	187
	D_1_		51.73	1342	89.59	162
	D_3_		16.72	1518	39.74	118
	G		21.37	1599	81.77	73

**Table 3:** Results summarizing the deconvolution of C1s, O1s, Bi4f, and Fe2p_3/2_ experimental XPS spectra recorded for XGAr and XGN nanocomposites.

C1s									
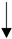	Sample	Component 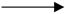	Defects	C=C sp2	C-C sp3	C-OH C-O-C	C=O	O=C-OH	π- π* Shake-Up
XGAr		B.E. (eV)	283.3	284.5	285.6	286.6	287.8	288.9	290.3
		%	1	73.5	12.3	6	3	3	1.3
XGN		B.E. (eV)	283.3	284.5	285.5	286.5	287.7	288.9	290.3
		%	1.1	72	11.4	6.8	3.3	3.3	2.1
**O1s**									
**Sample**			**Component**		**Fe-O, Bi-O**		**OH**	**C-O**	**O-C=O**
XGAr			B.E. (eV)		530.3		531.8	532.9	533.8
			%		36.6		29.7	20.6	13.1
XGN			B.E. (eV)		530.4		531.9	533.1	534.3
			%		35.5		32.8	24.7	7
**Bi4f**									
**Sample**			**Bi4f Component**		**Bi^3+^**		**Bi^0^**		
**XGAr**			B.E. (eV)		4f_7/2_	4f_5/2_	4f_7/2_	4f_5/2_	4f_5/2_
			%		159.3	164.6	157.2	162.5	162.5
XGN			B.E. (eV)		50.2	40.7	5.2	3.9	4.2
			%		159.2	164.5	157.1	162.4	162.4
**Fe2p_3/2_**									
**Sample**		**Component**	**Fe^0^**	**Peak1**	**Peak2**	**Peak3**	**Peak4**	**Peak5**	**Surface Structures**
XGAr		B.E. (eV)	707.28	709.78	710.53	711.44	712.34	713.48	714.37
		%	5.6	10.77	25.30	27	13.85	10.36	7.09
XGN		B.E. (eV)	707.20	708.90	710.20	711.28	712.34	713.48	714.87
		%	3.14	4.20	31.66	30.89	18.93	8.93	2.24

**Table 4:** The parameters of the equivalent circuit.

	Electrode		
	GC	GC/Chi-XGN	GC/Chi-XGAr
R_el_ (Ω·cm^2^)	5.40 ± 1.23	10.72 ± 6.67	7.58 ± 3.30
CPE_dl_ (µS·s^n^/cm^2^)	14.83 ± 5.6	327.9 ± 1.26	662.5 ± 6.82
n	0.80 ± 0.94	0.63 ± 3.38	0.72 ± 1.99
R_ct_ (Ω·cm^2^)	3706 ± 1.88	2432 ± 2.25	2624 ± 2.31
W (mS·s^1/2^/cm^2^)	1.53 ± 2.36	1.51 ± 2.70	1.70 ± 3.15
C (µF/cm^2^)	7.18	287.1	821.4
χ^2^	0.005918	0.003435	0.00148

**Table 5:** Analytical parameters of nonenzymatic sensors based on carbonaceous materials and iron (reported in the literature) used for detection of H_2_O_2_.

Electrode Type	Applied Potential V vs. Ag|AgCl,KCl_sat_	Linear Range	Detection Limit (µM)	Ref.
(Fe-CA)-CPE	−0.3	1–50 mM	500	[9]
GC/Chi-CXBiFe_0_	−0.3	1–10 mM	842.24	[12]
GC/Chi-CXBiFe_0.01_	−0.3	3–30 µM	0.85	[12]
GC/Chi-CXBiFe_0.12_	−0.3	3–30 µM	0.43	[12]
GC/Chi-CXBiFe_1.2_	−0.3	3–30 µM	0.24	[12]
α-Fe_2_O_3NS_|FePO_4_	−0.3	6–270 µM	3.4	[28]
Fe_3_O_4_/r-GO/GC	−0.3	20–280 nM	0.006	[29]
GC/RGO/Au/Fe_3_O_4_/Ag	0.55	0.002–1.2 mM	1.43	[30]
GC/Chi-XGAr	−0.3	1–10 µM	0.17	This work
GC/Chi-XGN	−0.3	1–10 µM	0.36	

GC, glassy carbon; Chi, chitosan; CX, carbon xerogel; CA, carbon aerogel; r-GO, reduced graphene oxide.

**Table 6:** Analytical parameters of the sensors based on XGAr and XGN nanocomposites and other carbonaceous materials (reported in the literature) used for SWASV detection of Pb^2+^.

Electrode Type	Peak Potential V vs. Ag|AgCl,KCl_sat_	Linear Range	Sensitivity (µA/µM)	Detection Limit (pM)	Ref.
GC/Chi-(Bi-CX)	−0.55	1–10 pM	1.15·10^6^	0.36	[11]
GC/Chi-(Bi-CX)	−0.56	1–10 pM	1.37·10^6^	0.28	[10]
GC/Chi-(Bi-CA)	−0.44	1–10 pM	2.3·10^5^	0.48	[10]
GC/Chi-CXBiFe_0_	−0.53	1–10 pM	1.17·10^6^	0.36	[12]
GC/Chi-CXBiFe_0.01_	−0.58	1–10 pM	1.01·10^6^	0.54	[12]
GC/Chi-CXBiFe_0.12_	−0.56	1–10 pM	3.77·10^5^	0.77	[12]
GC/Chi-CXBiFe_1.2_	−0.51	1–10 pM	6.39·10^5^	1.24	[12]
Bi/Fe_2_O_3_/G/GC	−0.6	4.8–480 nM	0.08	338.16	[31]
Fe_3_O_4_/MWCNTs/GC	−0.63	20 pM–1.6 nM	1.14·10^4^	6	[32]
Fe_3_O_4_/rGO GCE	−0.6	0.4–1.5 µM	13.6	169·10^3^	[33]
PVA/chitosan-TRG/GC	−0.58	4.8–241.3 nM	-	241	[34]
GC/Chi-XGN	−0.46	0.1–1 nM	8.44·10^3^	33.05	This work
GC/Chi- XGAr	−0.42	0.1–1 nM	6.47·10^3^	46.36	

**Table 7:** Operational stability of Pb^2+^ and H_2_O_2_ detection at GC/Chi-XGAr and GC/Chi-XGN modified electrodes.

Electrode	Analyte	Peak Potential		Current	
		V vs. Ag/AgCl,KCl_sat_	RSD (%)	µA	RSD (%)
GC/Chi-XGAr	Pb^2+^	−0.414 ± 0.007 *	1.84 *	4.97 ± 0.02 *	0.42 *
	H_2_O_2_	-	-	2.807 ± 0.05 **	1.88 **
GC/Chi-XGN	Pb^2+^	−0.464 ± 0.005 *	1.26 *	5.84 ± 0.01 *	0.26 *
	H_2_O_2_	-	-	1.396 ± 0.03 **	2.32 **

* SWV results averaged over 3 measurements on the same modified electrode immersed in 0.1 M acetate buffer (pH 4.5) containing 0.7 nM Pb^2+^; ** amperometry results averaged over 3 measurements on the same modified electrode immersed in 0.1 M phosphate buffer (pH 7) containing 15 µM H_2_O_2._

## Data Availability

Dataset available on request from the authors.
